# Patient perspectives on the ethics and acceptability of perfusion techniques for organ transplantation: a qualitative study

**DOI:** 10.3389/ti.2026.16459

**Published:** 2026-07-08

**Authors:** Jaden Blazier, Eline M. Bunnik, Valerie Kortekaas, Esther W. de Bekker-Grob, Maartje H. N. Schermer, Niels van der Kaaij, Robert Minnee, Olivier Manintveld, Jeroen De Jonge, Emma K. Massey

**Affiliations:** 1 Department of Health Technology Assessment, Erasmus School of Health Policy and Management, Erasmus University Rotterdam, Rotterdam, Netherlands; 2 Department of Internal Medicine, Erasmus MC Transplant Institute, Erasmus MC, University Medical Centre Rotterdam, Rotterdam, Netherlands; 3 Department of Public Health, Program of Medical Ethics, Philosophy, and History of Medicine, Erasmus MC, University Medical Centre Rotterdam, Rotterdam, Netherlands; 4 Department of Philosophy and Religious Studies, Ethics Institute, Faculty of Humanities, Utrecht University, Utrecht, Netherlands; 5 Department of Health Law and Ethics, Erasmus School of Health Policy and Management, Erasmus University Rotterdam, Rotterdam, Netherlands; 6 Division of Cardiothoracic Surgery, Erasmus MC Transplant Institute, Erasmus MC, University Medical Centre Rotterdam, Rotterdam, Netherlands; 7 Division of HPB/Transplant Surgery, Erasmus MC, University Medical Centre Rotterdam, Rotterdam, Netherlands; 8 Department of Cardiology, Erasmus MC, University Medical Centre Rotterdam, Rotterdam, Netherlands

**Keywords:** ethics, machine perfusion, normothermic regional perfusion, organ transplantation, patient perspectives, donation after circulatory death, qualitative research

## Abstract

Perfusion technologies preserve and/or improve organ viability by restoring circulation to donor organs either outside the body (machine perfusion, MP) or inside the body of the deceased donor (normothermic regional perfusion, NRP). Ethical debates surrounding perfusion raise questions about its acceptability for transplant recipients. This study explores patient perspectives on perfusion techniques and preferences regarding informed consent. Five online focus groups were conducted with 32 kidney, liver, and heart patients, both pre- and post-transplant. Sessions were analyzed using qualitative content analysis in Atlas.ti. Participants were generally accepting of MP and NRP, valuing clinical benefits. However, they were more hesitant toward NRP due to ethical concerns about post-mortem use of the donor’s body and potential emotional impacts on donor families. Drivers of acceptance were patients’ medical urgency and certainty that donor and donor family were respected. Most participants wanted to receive detailed information about perfusion techniques. While patients were broadly accepting of perfusion techniques, NRP raised more concerns than MP. To ensure ethical implementation and social acceptability as these technologies evolve, patient perspectives should be embedded into perfusion guidelines, informed consent processes, and policy development.

## Graphical Abstract

**Figure:**
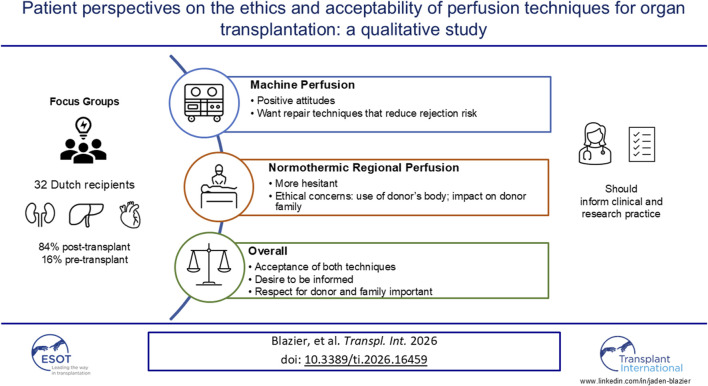

## Introduction

In response to persistent organ shortages, perfusion techniques are utilized to preserve, assess, and/or enhance organ viability by circulating blood or blood substitutes through donor organs, increasing the availability of organs [1, 2]. There are two perfusion techniques: machine perfusion (MP), performed *ex situ* outside the donor’s body, and normothermic regional perfusion (NRP), performed *in situ* within the donor’s body. In NRP, after the donor is declared dead based on circulatory criteria and the legally required no-touch period is honored, extracorporeal membrane oxygenation is used to reinstitute circulation to a targeted region in the donor’s body [3]. In TA-NRP, the heart resumes beating. Cerebral vessels are occluded with the aim of preventing reperfusion of the brain during NRP, although there is a theoretical minor risk of collateral blood flow to the brain through spinal arteria [4], which fortunately has not been substantiated in recent research [5, 6]. While both techniques have been shown to improve transplant outcomes over traditional static cold storage without significant differences in outcomes between techniques [7–9], they have distinct advantages and drawbacks. For example, MP offers longer preservation times, but can be more costly, while NRP enables multi-organ perfusion and *in situ* functional assessment [10–12].

The different perfusion techniques also present distinct ethical issues [13]. Critics have argued that NRP negates the determination of death and/or violates the dead donor rule by restoring the donor’s circulation, restoring heartbeat in TA-NRP, and/or risking restored brain circulation or function [14, 15]. MP avoids issues related to death determination, but it is more resource and labor intensive [16]. These concerns have led to varying adoption of both NRP and MP across countries, and deliberation about implementation continues [17, 18]. In Belgium and the UK, for instance, MP and A-NRP are utilized, but TA-NRP activity has been paused [19, 20]. In countries performing MP and/or NRP, the degree to which patients are informed or have a choice about the use of perfusion techniques varies by patient group, center, and country [16, 20–22].

In current practice, MP and NRP facilitate the utilization of organs which otherwise would not have been deemed suitable for transplantation via better preservation and viability testing. While early clinical data suggests that these organs–which have been classified by some centers as “high risk”–can function as well or better than standard organs after perfusion [23], long-term clinical data is not available yet. In the future, MP also presents the opportunity to deliver novel therapies to repair organs *ex situ*, e.g., regenerative stem cell therapies or immunomodulation [1], but these applications could also pose unforeseen risks.

The debate about the ethicality of perfusion techniques raises questions about their acceptability and how much information organ recipients and other stakeholders should receive about perfusion. While there have been calls for qualitative research on these issues [13], empirical research into the perspectives of organ recipients, or their families, on perfusion has been sparse and limited to Canada [24–26]. (Potential) recipients are key stakeholders because the manner in which organs are procured and preserved could have moral, emotional, and clinical significance to the recipient, who will ultimately receive the organ. Understanding their attitudes, concerns, and preferences can guide clinical implementation and ensure ethical legitimacy of perfusion techniques.

To address this gap, this qualitative study was designed to explore the perspectives of transplant candidates and organ recipients (hereafter: patients) on perfusion technologies, with specific attention to ethical issues. We aimed to understand: 1) their perspectives on and acceptance of perfusion techniques and 2) their preferences surrounding informed consent and future applications.

## Materials and methods

### Study design

We conducted online focus groups with Dutch transplant patients. In contrast to interviews, focus groups enabled participants to engage with each other to develop their ideas, ideal for discussing controversial or unfamiliar techniques [27]. The Netherlands is an important context for investigation due to its early adoption of perfusion techniques in Europe [15] and potentially differing cultural attitudes compared with previously studied Canadian cohorts [19, 20]. Online sessions allowed participants from across the Netherlands to participate. The study received ethical approval from the Erasmus Medical Center (Erasmus MC) Medical Ethics Review Commission. Written informed consent was collected from all participants. Our methods are reported in accordance with the Consolidated Criteria for Reporting Qualitative Research (see Supplementary Appendix 1) [28].

### Sampling and recruitment

We conducted purposive recruitment of participants from three patient groups (kidney, liver, or heart) who were awaiting or had received a transplantation, who were above 18 years old, and spoke Dutch. Advertisements were distributed by the Dutch Kidney Patient Society (NVN; nierstichting.nl), the Dutch Liver Patient Society (NLV; leverpatientenvereniging.nl), and the Erasmus MC Transplant Institute (erasmusmc.nl/en/transplantinstitute), via email and social media (LinkedIn, Instagram). A cardiologist handed out flyers during outpatient visits at Erasmus MC. Interested participants were emailed an online questionnaire (Qualtrics) to collect demographic and clinical information. We attempted to recruit a diverse sample of participants, and within each patient group, maximum variation sampling was utilized based on age, gender, cultural group, education level, and transplant status.

### Data collection

Semi-structured focus groups of 100–120 min were held online through a secure platform (Microsoft Teams) between 04/25 and 05/25. The sessions were facilitated in Dutch by a moderator (Author 1) with assistance from Author 3 and/or Author 2. An interview guide was utilized to probe participants with open-ended questions addressing several topics: general knowledge of perfusion; perspectives and acceptability; informed consent; and future applications (see Supplementary Appendix 2). Participants were shown a presentation with information about each technique and “communication scenarios” to ensure sufficient background information and to elicit responses (Table 1). The interview guide and presentation were developed by the broader research team based on a comprehensive literature review that identified key ethical debates surrounding MP and NRP [13]. Topics and questions were adapted from prior qualitative studies on MP and NRP [24–26] and patient attitudes towards novel organ transplant interventions [29–31]. The “communication scenarios” included three hypothetical approaches which we extrapolated from the debate in the literature on informing stakeholders about perfusion [13]: obtaining informed consent for perfusion, disclosing that perfusion is used, or not disclosing the use of perfusion. The interview guide and presentation were pilot tested with researcher colleagues in 3 rounds (n = 14) and feedback improved the clarity, neutrality, and accessibility of the information and questions. All focus groups were audio and/or video recorded and auto-transcribed verbatim, and each transcript was manually checked by two researchers (JB and VK, MG, or JZ). Data saturation was reached during data collection after four focus groups, meaning new themes did not arise with additional focus groups, and after one more focus group, recruitment was stopped.

**TABLE 1:** Communication scenario to elicit patient preferences on informed consent procedures for perfusion during focus groups.

Item	Content
Communication scenario	When your doctor is discussing organ transplantation with you and wants to place you on the waitlist… A. The doctor informs you about perfusion and you can choose if you would be open for receiving perfused organs in addition to standard organs B. Information is given about perfusion as a normal part of the transplantation procedure C. The doctor does not mention perfusion
Guiding questions on informed consent preferences and needs	⁃ Which of these procedures would you prefer, and why? ⁃ Does your answer differ for machine perfusion vs. normothermic regional perfusion? ⁃ What information would you need in order to make a good decision about the use of perfusion techniques? ⁃ In which way would you like to receive this information?

### Data analysis

Focus groups were thematically analyzed using qualitative content analysis, a method in which codes (labels about content) and themes are inductively derived from qualitative data [32, 33]. Data was managed in Atlas.ti. Five researchers (JB, EMB, EKM, MS, JS) independently open-coded one transcript and developed a codebook through consensus discussion. Two researchers (JB and EKM or VK) coded two more transcripts and refined the codebook. JB then coded the remaining transcripts and iteratively consolidated codes into themes in consultation with EKM and EMB. Themes are summarized in the text, and for themes that were more complex, sub-themes and representative quotes (manually translated to English) are presented in tables.

## Results

**FIGURE 1:**
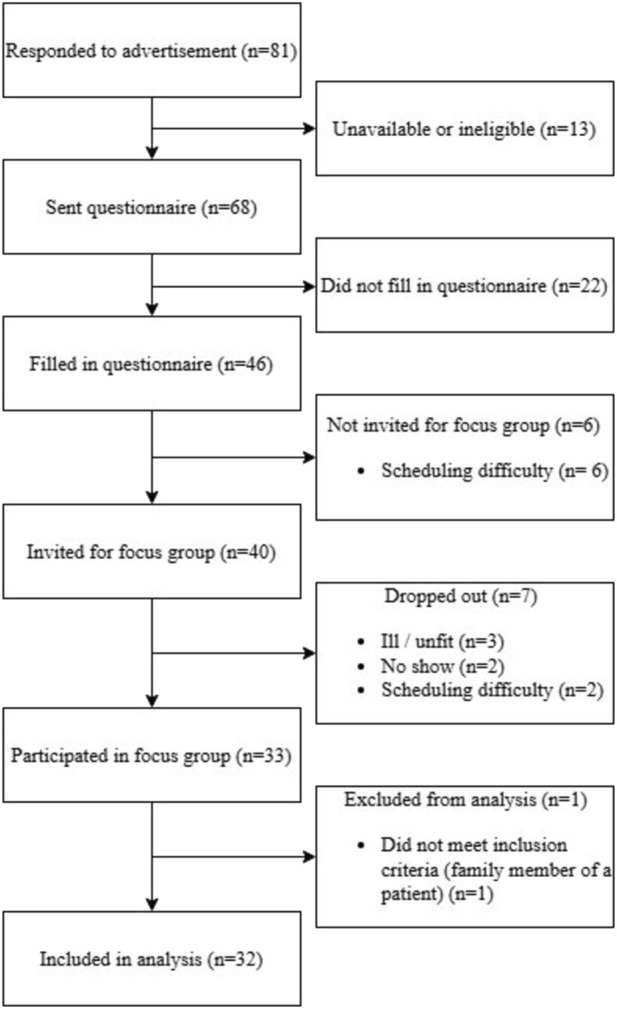
Flowchart of participant inclusion. The number of respondents that participated or dropped out at each point in recruitment for focus group study of patient perspectives on perfusion technology for organ transplantation, and the reasons.

Five focus groups were conducted (1-2 per organ type) with 3-9 participants each. 32 participants were included: 16 liver patients, 13 kidney patients, and three heart patients. Figure 1 shows the inclusion flowchart. Table 2 shows the demographic information of the participants. Almost all participants were Dutch (97%) and more than half were Atheist (53%). Most of the participants had received a transplant (84%); the rest (16%) were either on the waitlist or expecting to be waitlisted for a transplant shortly. Nine of the 13 kidney patients had been on dialysis, for an average of 3.8 (SD, 2.6) years. All three of the heart patients were on durable left-ventricular assist device support before being transplanted.

**TABLE 2:** Demographics of participants included in qualitative study on organ perfusion (n = 32).

Characteristic	Category	N (%)
Total participants	​	32 (100.0)
Patient group	​	​
​	Liver patients	16 (50.0)
​	Kidney patients*	13 (40.6)
​	Heart patients	3 (9.4)
Age (years)	​	​
​	18–35	1 (3.1)
​	36–55	16 (50.0)
​	56–75	13 (40.6)
​	76+	2 (6.3)
Gender	​	​
​	Woman	14 (43.8)
​	Man	18 (56.3)
Education level	​	​
​	Secondary education (high school)	4 (12.5)
​	Secondary vocational education	6 (18.8)
​	Lower professional education	0 (0.0)
​	Higher professional education (bachelor)	18 (56.3)
​	University: Bachelor	0 (0.0)
​	University: Master or higher	4 (12.5)
Employment status	​	​
​	Paid employment, full time	5 (15.6)
​	Paid employment, part time	4 (12.5)
​	Unpaid employment, full time	1 (3.1)
​	Unpaid employment, part time	1 (3.1)
​	Unemployed	21 (65.6)
Religion	​	​
​	Atheism/None	17 (53.1)
​	Christianity	11 (34.4)
​	Christianity: Jehovah’s witness	1 (3.1)
​	Other (e.g., Humanism)	3 (9.4)
Cultural group (self-identified)	​	​
​	Dutch	31 (96.9)
​	Surinamese Dutch	1 (3.1)
Donor registration status	​	​
​	“Yes, I want to become a donor”	28 (87.5)
​	“No, I do not want to become a donor”	2 (6.3)
​	“My partner or family decides after my death”	0 (0.0)
​	No objection (no active choice made)	1 (3.1)
​	I do not know my status	1 (3.1)
Transplant status	​	​
​	Post-transplant: one transplantation	17 (53.1)
​	Post-transplant: more than one transplantation	6 (18.8)
​	Post- and pre-transplant: on waitlist	2 (6.3)
​	Post- and pre-transplant: not yet on waitlist	2 (6.3)
​	Pre-transplant: on waitlist	3 (9.4)
​	Pre-transplant: not yet on waitlist	2 (6.3)
Time on waitlist (years)	​	Mean (SD)
​	Post-transplant: no longer on waitlist (wait time for latest transplant) (n = 25)	2.1 (2.2)
​	On waitlist at time of study (n = 5)	2.5 (3.1)
Time since last transplantation (years)	​	Mean (SD)
​	Post-transplant patients with correct transplant date data (n = 24)**	10.2 (9.4)

* One kidney patient also had a liver transplant.

** 3 values for transplant date missing: participants gave a date in the future or no date.

We identified the following six themes: 1) attitudes and perceptions; 2) perceived advantages; 3) concerns; 4) factors influencing acceptance; 5) informed consent preferences; and 6) future developments.

### Attitudes and perceptions

General attitudes toward perfusion technology were mixed, and varied by technique. Many of the participants had some prior knowledge about MP, but most had never heard about NRP. Only a few patients were aware that they had personally received a perfused organ, and all of these were liver patients. Participants’ attitudes toward MP tended to be positive, using words like “fantastic” and “beautiful.” Initial attitudes toward NRP tended to be more negative, some stating that NRP felt, “heavy,” “not good,” or “a bit Frankenstein.” Some took a more neutral stance, e.g., seeing TA-NRP as similar to donation after brain death. Some participants felt hesitant, finding it difficult to form an opinion about perfusion techniques. Technical questions were raised, such as why NRP would be used rather than MP?

### Perceived advantages

Participants tended to focus on the clinical advantages of MP, such as increased organ availability, objective testing, and increased matching possibilities through longer-distance transportation (see Table 3). Some noted that preservation might be better with NRP because the organ remains in its natural environment. They discussed how these advantages emotionally benefit patients; perfusion offers hope to waitlisted patients, and preserving an organ in a machine can create a safe feeling for recipients.

**TABLE 3:** Participants’ perceived advantages of and concerns about perfusion techniques, discussed by transplant candidates and recipients (n = 32) during focus groups.

Category	Perceived advantages	Example quotes	Concerns	Example quotes
Clinical or technical	⁃ MP: Increased availability of organs; use of previously unsuitable organs ⁃ Better quality organs due to preservation and repair ⁃ NRP: Natural environment for the organ ⁃ MP: Accurate/objective decisions due to viability testing ⁃ MP: Longer preservation; transportation to further geographic regions; better matching possibilities ⁃ MP: Plannable surgery; staff can perform better	“And at the moment that you then have more organs, in total, so to say, with which you can cover a longer distance [with MP], that you can tide over a longer time, then you are actually in a position, through those aspects, to make more optimal matches.” *P28, kidney patient, pre-transplant* “I think that inside the body [NRP] also has advantages, because you maintain everything in its own environment for a while.” *P23, kidney patient, pre-transplant*	⁃ Novel technique; there might not be enough knowledge about the technique and the long-term outcomes ⁃ MP: Organ might function less well than standard organ; complications due to perfusion; higher chance of rejection ⁃ MP: Third-party donor blood might lead to blood-group matching issues; risk of rejection ⁃ MP: Risk of technical failure ⁃ NRP: More potential risks and less clinical experience than MP ⁃ NRP: Donor’s decaying body might negatively impact organs	“[I]t is nerve-wracking, eh? What that [an MP organ] is going to do in the long term, eh?[…] Look, the experiences with the normal transplantations, those are there. And with the perfusion livers, the experiences with those are, so to say, still not clear.” *P7, liver patient, post-transplant* “[I]f you do that [NRP] in the deceased body, then you also have to deal with all sorts of aspects of decomposition and things like that. And I wondered[…] what possible harmful side effects that could have [on the organs].” *P28, kidney patient, pre-transplant*
Related to (potential) recipient	⁃ MP: More hope for waitlisted patients ⁃ MP: Less disappointment for potential recipients from transplant not occurring ⁃ MP: Safer feeling for recipient ⁃ MP: Plannable surgery; less stress for recipient	“[T]he so-called ‘unsuitable organs’ can now be used. So yeah, I think that this can only be very positive.” *P3, liver patient, post-transplant* “[B]ecause they can also improve the quality further [with MP][…] that really gives[…] a safer feeling in some way.” *P13, liver patient, post-transplant*	⁃ MP: Potential recipient might be disappointed if organ not suitable for transplant after viability testing ⁃ MP: Use of perfusion might impact recipient waitlist position, depending if they agree to perfusion or not ⁃ Potential religious/spiritual concerns or objections	“[B]ut, there can be religious convictions [against MP], I do not know.” *P21, kidney patient, post-transplant*
Related to donor (ethics)	⁃ MP: Less invasive and ethically complex than NRP ⁃ MP: Respects donor by perfusing only isolated organs ⁃ NRP: Respects donor by allowing slower actions and less unnecessary organ removal ⁃ Reduced need for living donors	“I think that as long as the warm perfusion still takes place within the body [NRP], and so, also all the first tests take place [within the body], it could be that it [the organ] is not taken out of the body. For some relatives, that can maybe also help, to know that it was not for nothing.” *P4, liver patient, post-transplant* “But I would still feel guilty about it [living donation]. Like, yes, that person has lost their kidney[…] And if that quality [of the MP organ] comes close to that of a living [donor] kidney anyway, through the perfusion via the machine, then yes, I would consider that ideal.” *P24, kidney patient, post-transplant*	⁃ NRP: Donor is both dead and alive ⁃ NRP: Donor’s circulation restarts ⁃ TA-NRP: Donor’s heart restarts ⁃ NRP: Donor’s body continues “going” ⁃ NRP: Length of time perfusion is carried out ⁃ NRP: Natural death process interrupted ⁃ NRP: Consciousness/soul might return ⁃ NRP: Brain activity might remain ⁃ NRP: Instrumentalization of donor’s body (using the body as a “machine”) ⁃ NRP: Manipulability of donor’s body ⁃ NRP: Donor might not have been informed; NRP is not part of what “donorship” entails ⁃ Potential religious/spiritual concerns or objections	“[I]f you then get things going again [via NRP], then that feels, from an ethical perspective, a bit like keeping it [the body] alive.” *P13, liver patient, post-transplant* “For me, there *is* a boundary somewhere, at the moment that you [are not only talking about parts of the body, but] can also, say, get at the consciousness, or sensation, or the soul of people, [through NRP], yeah, then it gets very, very murky for me.” *P28, kidney patient, pre-transplant* “You do see now, then, [through NRP], it seems a person becomes a sort of machine.” *P10, liver patient, post-transplant* “[T]he question is where the ethics is at, because it appears as if, using various means, you can enable, disable, temporarily suspend various components [of the body] and so on[…] It all raises some questions[…] how far do we go?” *P23, kidney patient, pre-transplant*
Related to donor family	⁃ MP: Respects donor family by perfusing only isolated organs and returning the donor’s body to the family ⁃ NRP: Respects donor family by allowing slower actions and less unnecessary organ removal	“Yeah, it feels like it [MP] would be better for the relatives [than NRP], because if you have machine perfusion, the body is available for the relatives again earlier. As long as it [the body] is still there on the warm perfusion [NRP], so still on the machine, the body is still not with you. If the organs are taken out of the body, and they are then placed in the perfusion machine, then the body is returned to the relatives again.” *P3, liver patient, post-transplant*	⁃ NRP: Emotional impact on donor family ⁃ NRP: Process of saying goodbye and grieving might be disrupted, e.g., by longer perfusion ⁃ NRP: Donor family might believe that the donor is still alive ⁃ NRP: Donor family might have false hope that the donor will recover ⁃ Potential religious/spiritual concerns or objections	“I can imagine that that [NRP] is, maybe, somewhat more intense for the relatives, but that is my feeling about it[…] because as long as they keep working with such a body, and a part of that body, so yeah, ‘continues to live…” *P31, heart patient, pre-transplant* “I mainly see disadvantages for the relatives of the deceased. [With NRP] it takes even longer before, yeah, the body is finally dead. When do you say goodbye? When is it over and done? It seems difficult to me.” *P26, kidney patient, post-transplant*

MP, machine perfusion; NRP, normothermic regional perfusion; P, participant; TA-NRP, thoracoabdominal normothermic regional perfusion.

Advantages for the donor and their family were also discussed. Some participants saw MP as more respectful to the donor and their family because only isolated organs are perfused, and the body is returned to the family without further intervention. Yet, some participants mentioned that NRP could help convey respect by slowing down the procedure, avoiding unnecessary organ removal if viability testing found organs to be unusable.

### Concerns

Several concerns about perfusion technology were raised by participants (see Table 3). First, clinical concerns centered around the novelty of perfusion techniques. Some participants worried about unknown long-term outcomes and risks, especially for organs that would have been deemed unsuitable for transplant had perfusion not been used. Many participants thought NRP was riskier than MP; some worried that decaying processes in the deceased donor’s body might damage the organs during NRP.

Second, ethical concerns related to the donor were raised, particularly for NRP. Some participants thought restarting circulation “keeps part of the body alive,” or interrupts the natural process of death. The heart restarting during TA-NRP was more troubling to some participants than abdominal circulation during A-NRP, for instance because the heart indicates life, while others saw no distinction between A-NRP and TA-NRP. Some said that they would only be concerned if consciousness or a soul would return to the body, but most did not personally believe this would occur, often presuming that donors undergoing NRP were irreversibly brain dead. Some remained concerned that parts of the body continue “going” after death, and that body parts can be enabled or disabled at will. Some described this aspect as using a human “as a machine.” Many participants thus had questions about the length of time that NRP would be carried out, which they thought should be limited, expressing acceptable durations ranging from one to ∼24 h.

Third, participants emphasized that NRP would primarily impact the donor’s family emotionally. They worried that the use of the body for NRP and/or relatives’ feeling unsure about the donor’s death determination might disrupt the process of saying goodbye, grieving, and arranging a funeral.

Finally, participants imagined that recipients, donors, or their families might have religious objections to MP and NRP, but did not specify these further.

### Factors influencing acceptance

Despite their potential concerns, most participants stated that they would still personally accept any organ offered to them, regardless if either perfusion technique was used. The factors that might influence willingness to accept a perfused organ were discussed (see Table 4). First, clinical utility was relevant to participants. Many were mainly concerned whether the organ works, for instance, that it “meets the requirements of the test” during perfusion. While experience and research indicating safe outcomes were important to some participants, others stated that individual outcomes can never be predicted, and they might still accept a perfused organ even if outcomes were statistically inferior to alternatives.

**TABLE 4:** Factors influencing participants’ willingness to accept perfused organs, discussed by transplant candidates and recipients (n = 32) during focus groups.

Category	Factors influencing acceptance	Example quotes
Clinical	⁃ Evidence of clinical outcomes; quality of organs compared to standard organs; impact on symptoms/quality of life ⁃ Level of experience/research about the technique; how common the technique is ⁃ Necessity of technique to achieve the aims ⁃ Degree to which physician supports the technique ⁃ Degree to which recipient believes individual transplant outcomes are unpredictable no matter the technique used	“And I personally feel, for me it would not matter if that [organ] came from living perfusion [NRP] or from a perfusion machine [MP][…] I think that it revolves around how good the organ is.” *P28, kidney patient, post-transplant* “Yeah, I am very simple in that. If it [the organ] meets the requirements of the [viability] test, then it meets the requirements, period.” *P30, heart patient, pre-transplant* “The patient is dead, yeah. If that [blocking the blood vessels to the brain during NRP] is the best method to do that, then you should do it.” *P30, heart patient, pre-transplant* “And that perfusion through the body [NRP], I just find it … I find it too vague, yes, at this moment. When there’s more experience with it [NRP], and when, yeah, there’s more insight into it, then that [my opinion] might change. But at the moment, I’m thinking, 'No, I would not want that.’” *P24, kidney patient, post-transplant*
Related to (potential) recipient	⁃ Medical urgency of recipient’s condition; amount of time pressure; availability of alternative options ⁃ MP: Quality of life of recipient ⁃ MP: Time spent waiting; time spent on dialysis ⁃ Perspective as a patient; impact of illness on the relevance of ethical limits for the patient ⁃ Age of recipient; relevance of long-term outcomes ⁃ Degree of trust in medicine; belief that the physician should decide about perfusion ⁃ Attitude of recipient towards new technologies ⁃ Degree to which recipient is informed about the perfusion procedure and aims	“Only, at the moment that you are on the [waiting] list as a liver patient, yeah, very honestly, then it is also life and death[…] I think that as a recipient, you are always happy with what you get, and in whatever way it comes.” *P13, liver patient, post-transplant* “[I]f someone says to me, yeah, ‘the [perfused] liver is not completely good, we do not know for sure,’ then I say, ‘go ahead.’ because the alternative is that I might die, so in any case I do not think that, if they have doubts, that they should put that on the patient.” *P12, liver patient, post-transplant* “So I think that everyone that has ethical or moral objections towards[…] all kinds of techniques that are used, will immediately throw them overboard the moment that it is your turn and your life can be saved. It’s that simple, in my opinion.” *P4, liver patient, post-transplant*
Related to donor (ethics)	⁃ NRP: Degree to which donor is treated with respect ⁃ NRP: Whether the donor has given informed consent to the procedure ⁃ NRP: Degree to which harm to donor is prevented ⁃ NRP: Degree of certainty that donor is (brain) dead ⁃ NRP: Length of time perfusion is carried out ⁃ Procedure’s accordance with laws	“[F]or me, it would not be such a big problem if the heart had to be kept beating [during NRP], just as long as it would be clear that there was no chance of recovery, and that the person was really brain dead.” *P7, liver patient, post-transplant* “[J]ust, it [perfusion] has to happen in a correct [and fair] manner[… f]or both the donor and for the recipient. That it is done in a neat and orderly way, so that everyone knows what has happened.” *P32, heart patient, pre-transplant* “[The need for respect also applies] if someone registers as a donor, so then you make your organs available for further use. But you do not realize that they’re still working with your body for a certain amount of time. So I wonder, is that perfusion [NRP] actually a part of donor-ship, as everyone understands it?” *P15, liver patient, post-transplant*
Related to donor family	⁃ NRP: Degree to which donor family is treated with respect ⁃ NRP: Whether donor family has given informed consent to the procedure	“If it is common knowledge that this is how it happens [blocking the blood vessels to the brain during NRP], and the donor and the family accept that, then yeah, that is fine with me too.” *P31, heart patient, pre-transplant* “I think, the better you explain to the relatives what you are doing and *why* you are doing that, and also support them through that[…] then they also have a better feeling about the process. I think[…] that that is the most important.” *P3, liver patient, post-transplant*

MP, machine perfusion; NRP, normothermic regional perfusion; P, participant; TA-NRP, thoracoabdominal normothermic regional perfusion.

Second, the (potential) recipient’s physical health would play a large role in their willingness to accept perfused organs. Many participants, especially liver and heart patients, emphasized that waitlisted patients “have no choice” due to medical urgency and lack of alternative treatment options. Because desperation would lead them to accept any organ, some believed that the physician should decide whether to transplant a perfused organ or not. Participants felt that desperation would be increased by lower quality of life, longer time on the waitlist, and longer time on dialysis. Some claimed that when a patient faces death, their ethical boundaries fade or disappear. Many participants thus speculated that the perspectives of the general public or donors’ family members on ethical acceptability would differ from those of (potential) recipients.

Finally, some participants emphasized the importance of respecting both donors and their families during NRP, including proper information delivery and consent from all parties, confirmation of (brain) death, absence of harm, and compliance with laws. Others viewed ethical concerns related to the donor as less influential on their decision to accept a perfused organ, given the lack of a personal relationship with the donor.

### Informed consent preferences

Informed consent preferences were discussed on the basis of the “communication scenarios” shown to participants (Table 1). Table 5 lists the reasons they gave for their preferred informed consent procedures (A, B, or C). Most participants chose B: to be informed about perfusion. Some preferred A: to have the choice about accepting perfused organs or not. Some reasons for choosing A or B were that perfusion differs from standard donation, that risks might need to be weighed, and that some might have religious or ethical objections. Some did not think it was important to have a choice about perfusion, stating that B or C would be acceptable, e.g., because they would accept any organ offered.

**TABLE 5:** Participants' preferred informed consent procedures for perfusion.

Reasons given by participants for their responses during focus groups	Preferred consent procedure: “When your doctor is discussing organ transplantation with you and wants to place you on the waitlist…”			Example quote
	A: The doctor informs you about perfusion and you can choose if you would be open for receiving perfused organs in addition to standard organs	B: Information is given about perfusion as a normal part of the transplantation procedure	C: The doctor does not mention perfusion	
Freedom of choice is important; the patient has a desire to weigh the risks and benefits themself	A	​	​	“Yeah, I think that you should always have the freedom of choice [about perfusion]. I was also asked, ‘Do you want [an organ from] someone who had hepatitis, or not?’ Yeah, then I was also faced with a choice.” *P13, liver patient, post-transplant*
The perfusion procedure differs from standard donation	A	​	​	“I also think that the choice [about the use of MP or NRP] should be communicated [to the patient] in advance[…] these are all techniques that are used which deviate from the normal, and the—, yeah, what is normal about a donation? But, for a donation, normal is: out of the body, into the body.” *P16, liver patient, post-transplant*
Transparency is important; it would be unethical not to inform patients	A	B	​	“Yeah, I would just say B. I think that the doctor has to inform you about the possibility that perfusion is carried out as a part of the program, because I do find it important that you know what has happened to that organ. But yeah, I assume that the doctor knows what he is doing, right?” *P7, liver patient, post-transplant*
There is a relevant choice to be made; outcomes or risks might differ between the various options	A	B	​	“But I think it’s also very important here: What are the advantages and disadvantages? Because what I understand is that what’s causing me a lot of trouble right now, which is my bile ducts, is probably the consequence of perfusion[…] And in that case, if you were given the choice… I would now – but that’s only knowing what I know now and to what extent it affects my life – now, I would not choose perfusion.” *P4, liver patient, post-transplant*
There should be the possibility for objections (religious/conscientious/ethical)	A	B	​	“I think that this question [asking the patient if they agree with the use of perfusion] belongs with the transplant team before you go[…] onto the transplantation list, because I do find that important. Because if someone has a conscientious objection, or yeah, for example, sees medical ethical problems, then he has to say, ‘then I do not want to have [an organ] from such a person,’ and then it [the organ] goes to another person.” *P19, kidney patient, post-transplant*
Information gives a safe feeling and helps the patient prepare for the procedure	​	B	​	“For me, it was literally drawn out, ‘What is going to happen? What does the operation look like? But also, what will happen?’ Well yeah, so also about the perfusion, that was also spoken about[…] I am the person that does like to go into the operation well prepared[…] [I]t just helps with a feeling of safety.” *P13, liver patient, post-transplant*
Information is also given about other aspects of the transplant procedure, so one may as well also inform about perfusion	​	B	​	“Currently, a whole lot of things are also explained[…] also other things that are possible [during the transplant procedure]. So, I think that it is actually very logical that they can also just inform about this [perfusion], and informing is actually already asking.” *P28, kidney patient, post-transplant*
Waiting until the technique is no longer new before normalizing its use would be too long to wait (as with other technologies)	​	B	​	P30, heart patient, pre-transplant: “Because I get that everything is totally new, and you say, ‘Well, we have something new, we working on it. Are you interested in that?’ but, that is indeed a good question: where is that tipping point that we actually consider it [a transplant with perfusion] to be a normal form of transplantation?” P32, heart patient, pre-transplant: “Yeah, I think when the long term risks are fully mapped out. And that is of course— ” P30, heart patient, pre-transplant: “Yeah, but when is that then? 20 years? Then you have to wait 20 years… And that seems very long to me, with these sorts of, uh, developments[…] Yeah, because the ICD [implantable cardioverter defibrillator] that I now have is a newer version than the one that I had at first. And there was also never anyone that said to me, ‘Oh, we have a new one again, and yeah, this one has not yet been tested for 5 years, but we are just going to put it in.’”
Options can be overwhelming and complicate the process	​	B	​	“At a certain point, you can also give the patient too many choices, I think, eh?” […] I do think that you have to state it [that perfusion is used], also if it is a part of your standard procedures or possibilities that you have … Well, I would also not make too many requirements around it, because with that, you only make it more difficult, I think.” *P30, heart patient, pre-transplant*
The patient is in urgent condition, has time pressure, and/or has a lack of alternative options	​	B	C	“It would not matter to me if it would be B or C. Because it is a very privileged situation to be able to choose, if you have enough time, but a whole lot of people that are waiting for an organ here do not have that time. And I assume that perfusion, in any case, has the purpose of improving the quality of the organ. And then, that is good that you have that, but if you are in a hurry, it also does not matter anymore.” *P26, kidney patient, post-transplant*
The doctor should decide about perfusion; the patient is too ill and/or not medically informed enough to make a good decision	​	B	C	“I think it is sufficient to be well informed and to receive the information from your doctor, and I trust that he makes the right decision[…] And I do not want to get in the middle of that, as a patient like, ‘I choose for perfusion or not,’ because I am really just a layperson, not medically educated. So yeah, I think, B is already enough for me.” *P12, liver patient, post-transplant*
Outcomes from perfusion are the same or better than alternative options	​	B	C	“If it really did not matter if you would receive it [the organ] from the perfusion pump, from a warm perfusion [NRP], or a cold perfusion [MP], or from ice [static cold storage], if that all really would not make a difference [for the outcomes], then I would not need to know it [if perfusion is applied].” *P7, liver patient, post-transplant*

During focus groups, transplant candidates and recipients (n = 32) were shown the scenario with three possible options for communication procedures, and were asked to state their preferred procedure and why. Only some made distinctions between preferred communication procedures for NRP and MP, and in these cases they tended to prefer to have more information or more of a choice about NRP than MP. A, B, or C = at least one participant gave this reason for their answer during the focus group. MP, machine perfusion; NRP, normothermic regional perfusion; P, participant; TA-NRP, thoracoabdominal normothermic regional perfusion.

Table 6 lists the information that participants stated that they would like to know when making a decision about receiving a perfused organ. Some participants wanted the “complete picture” about how MP and NRP procedures are carried out, including all information presented in the focus group. Additionally, they wanted more information about clinical outcomes and risks. Participants wished to receive this information early in the transplant trajectory, to be able to decide how much information they receive, and to have access to a brief pamphlet or film. Some patients were also concerned about whether the donor’s family would be well-informed about the perfusion procedure, and added that families should receive specific information such as how long NRP would be performed, and when they could see their deceased relative again.

**TABLE 6:** Information about perfusion techniques that participants would like to receive prior to transplantation.

Topic	Specific information
Information about both techniques: MP and NRP	⁃ Advantages and disadvantages of each technique
What/how/why	⁃ What procedures are performed on the organ/donor ⁃ Type of perfusion used ⁃ Content of perfusate ⁃ How perfusion is carried out ⁃ NRP: Restarting circulation/heartbeat; blocking blood vessels to the brain ⁃ How organs are handled throughout the process ⁃ How organs are transported ⁃ Why perfusion is performed
Impacts on recipient	⁃ Risks and benefits ⁃ Statistics about clinical outcomes ⁃ Quality of organ offered ⁃ Impact on wait time for an organ ⁃ Impact on (blood-group) matching
Research and experience with perfusion	⁃ How long the technique has been used ⁃ If long term outcomes are unknown, how long is “long-term”? ⁃ In which countries the technique has been used
*Additional information for the donor family	⁃ Length of time of perfusion ⁃ When family members can see their deceased relative again

Transplant candidates and recipients (n = 32) stated during focus groups various topics regarding perfusion about which they would like to be informed, either at the moment of being registered for the waitlist, or sometime before the transplantation procedure.

* Participants specified that the donor family should also receive information about perfusion, including additional information that would be relevant to them. MP, machine perfusion; NRP, normothermic regional perfusion.

### Future developments

Finally, potential future applications of perfusion were discussed: repair of “unsuitable” organs with medicines, regenerative stem cell treatments, or genetic modification. Participants were generally open to organ repair techniques, one participant comparing it to his “refurbished iPhone[…] which functions perfectly.” However, when discussing their willingness to accept repaired organs in an early research phase in which outcomes are unknown, we observed more hesitancy. Some stated that they would only participate in an “experiment” if they were very old, or if it was their only possibility to survive.

Repair techniques were compared to alternatives, artificial organs and genetically modified animal organs, and several common preferences emerged. Participants tended to prefer techniques which would offer the most clinical utility, regardless of the source of the organ. They preferred techniques which would decrease the risk of rejection and the need for immunosuppressive mediation, such as regenerative treatments utilizing their own stem cells, or artificial organs. They tended to prefer human-sourced stem cells, tissues, and organs over those sourced from animals, some fearing risks like rejection or pandemics, or disapproving of animal cruelty. Finally, some participants were hesitant about genetic modification of organs, preferring modification to be restricted to minor repairs rather than enhancement, fearing unknown risks or finding it wrong to “go completely against nature.” A few participants stated that they would maintain certain ethical boundaries even at the expense of their own survival.

## Discussion

This qualitative study provides insight into the perspectives of kidney, liver, and heart patients (pre- and post-transplant) on acceptability and preferences regarding organ perfusion techniques. Participants were overall accepting of perfusion techniques, but raised more ethical concerns about NRP than MP. Nearly all participants thought recipients and donors’ families should be informed about the use of perfusion techniques, especially NRP. Prior qualitative studies on recipient (family) perspectives of either MP or NRP have only been performed in Canada [24–26]. These studies, similar to ours, found acceptance of perfusion amongst participants, facilitated by clinical utility, medical urgency, trust in physicians, and trust in the donor’s death determination. Our study expanded the investigation to the Netherlands, compared views on multiple techniques (MP and NRP), and elicited (reasons for) informed consent preferences. Three themes merit further interpretation and discussion.

First, while the patients in our study raised ethical concerns about NRP, they focused on different issues than those most commonly discussed in the literature. Typically debated among academics are the clamping of the donor’s cerebral vessels, the possible return of brain circulation or activity, and the negation of the death determination [34]. Even when prompted to share their views on cerebral vessel clamping and the risk of restored cerebral circulation, only a few participants raised concerns about these aspects. They were more concerned about NRP reanimating part of the donor’s deceased body and that NRP uses this body ‘as a machine,’ instrumentalizing the donor. These comments highlighted that, in NRP, the donor’s own circulatory system is utilized for the function of reperfusing organs, solely for the benefit of the recipient, seeming to compare this to the function performed by the perfusion machine, which some viewed as a potentially inappropriate use of the donor’s body. They argued that these aspects might in themselves be problematic for donors’ families, even if the donor is not truly revived or capable of suffering. Participants’ perspectives therefore diverged from common argumentation in the literature that NRP is concerning only if the donor is brought back to life in a meaningful way, e.g., if brain function is restored [35, 36]. Some participants did argue along these lines, but often presumed that donors are irreversibly brain dead during NRP, a presumption also observed amongst Canadian interviewees [25]. Meanwhile, novel concerns about NRP, such as instrumentalization, may deserve greater attention. Ensuring respect for the donor, so they are not treated merely as a means to an end, could help address these concerns. Empirical research on how respect can be conveyed to donors and their families during NRP could further strengthen respectful clinical practice and support ethical and social acceptability.

Second, some assumptions appeared to have influenced participants’ concerns about perfusion, but these indicate issues of importance to patients. First, some participants believed NRP would shorten the time for relatives to say goodbye after the death of their loved one, potentially unaware that standard DCD procedures offer the same period of time to say goodbye before organ procurement begins. Rather than a critique of NRP, this concern points to the impact of DCD procedures in general on the culture and rituals surrounding death [37]. Second, some believed that NRP would unacceptably extend the duration of time that the donor’s body is kept from their family. In reality, the additional one - two hours for NRP may be relatively little compared to an approximately three-hour DCD procurement procedure. However, if the duration of NRP is extended in the future, this concern should be taken seriously. Finally, some were concerned about the impact of the decaying body on organs during NRP. Although the anoxic brain releases toxic catecholamines, cerebral vessel occlusion and washout during perfusion limits their harmful effects [38]. While education and communication about the procedure could help correct some assumptions and foster acceptance, there will remain a plurality of beliefs about death and proper treatment of the deceased which are unlikely to be impacted through education about clinical elements [39, 40]. For example, some participants viewed death as a natural process that should not be disrupted, expressing concerns about both the unknown biological effects of NRP and a perceived violation of the sanctity of dying. Respecting these diverse beliefs requires adequate information provision to support individual decision making [14].

Third, our findings on informed consent procedures are valuable for developing guidelines for communication about perfusion with recipients and donor families. In current practice, centers may not inform patients about perfusion. Yet, our study contributes to a growing body of evidence that many patients want to know what procedures are performed on organs, especially procedures that diverge from standard care or could impose risks [24, 29], and NRP appeared to many participants to do so. While logistical barriers such as privacy laws could limit communication in practice, our findings support a baseline level of information delivery, which could be tailored on a case-by-case basis depending on individual patient desires and values. Some participants also wanted to be able to choose which perfusion techniques are applied. As participants pointed out, meaningful choice is limited when survival is at stake. But, greater patient involvement may be more important for those with alternatives like dialysis, and will become increasingly relevant as perfusion technologies evolve, availability expands, and risk levels diversify [21, 29]. Participants also cared that donors and their families were properly informed about perfusion, especially NRP, an important consideration for policymakers amidst debate about how transparent to be with these stakeholders [15, 41–43].

Contextual factors may influence the interpretation and transferability of our findings. First, the Dutch context differs in culture, values, and norms compared to other countries [44]. We observed a high degree of trust in physicians and the donor’s death determination, driving participants’ acceptance of perfusion techniques, which was also reflected in Canadian studies [24–26]. Yet, public surveys in North America have found resistance to perfusion techniques amongst minority groups with lower trust in the medical system [45, 46]. Robust involvement of stakeholders in decision making may be of more importance in contexts where trust in the medical system is lower or where autonomy is highly valued, reflected in calls for increased transparency about perfusion in the USA [21, 22, 41]. Additionally, the medical urgency experienced by transplant candidates could drive them to accept many techniques, even novel research interventions [30]. Yet, we found that some participants expressed limits to their acceptance of novel repair applications or alternatives like xenotransplant, which resonate with findings found in a UK public survey [47]. This highlights the need for policymakers to consider both patient and public views in future developments of perfusion technologies.

Limitations of our study were the small sample size, particularly for heart and pre-transplant patients, and the lack of religious and cultural diversity in the sample. Unspecified religious concerns were discussed by participants. Possible religious concerns have also been raised by ethicists [14] and by the Canadian general public in surveys [46]. Future research with a more purposively selected sample would be necessary to elucidate these concerns, and physicians ought to pay attention to religious or other concerns individuals might raise during informed consent procedures. Differences between patient groups of different organ types–for example, the impact of some patient groups having more alternatives available such as kidney dialysis, or distinctions between pre- and post- transplant patients–require a larger sample size to confirm. Further, despite attempts at neutrality and encouraging diverging opinions, we cannot fully rule out the influence of information framing [45] and social influences driving conformity in focus groups [48]. Finally, as pointed out by participants, registered donors and family members of deceased donors are likely to have unique concerns and information needs, which should be investigated, a gap also identified by others [17, 49].

Our study offers an in-depth exploration of the perspectives of transplant candidates and recipients who could receive a perfused organ, which are valuable for physicians and researchers working with perfusion techniques to understand. To ensure ethical implementation and societal acceptance, recipient preferences and moral opinions should be taken into account in decisions about clinical uses of perfusion techniques, informed consent procedures, and research on future developments.

## Data Availability

The pseudonymized focus group transcripts contain sensitive personal data from transplant recipients. Participants did not provide consent for re-use or sharing of their full transcripts beyond the original research team. In line with this consent and institutional privacy requirements, the transcripts cannot be made available to other researchers. Aggregated findings and selected anonymized/pseudonymized quotations are reported in the article. The raw, non-pseudonymized data cannot be made publicly available due to privacy constraints.
