# Supplementary material for: Patient perspectives on the ethics and acceptability of perfusion techniques for organ transplantation: a qualitative study
Source: Transpl Int. 2026 Jul 8;39:16459. doi: 10.3389/ti.2026.16459 (PMC13388232; doi:10.3389/ti.2026.16459)
Supplement: Supplementary file 1 [file Supplementaryfile1.pdf]

Appendix 1. COnsolidated criteria for REporting Qualitative research (COREQ) Checklist

Topic	Item No.	Response
**Domain 1: Research Team and Reflexivity**		
Personal Characteristics		
Interviewer/facilitator	1	Primary interviewer: JB<br>Secondary interviewer: VK, EMB
Credentials of interviewer	2	JB: MA, BSc<br>VK: MA, BA<br>EMB: PhD
Occupation of interviewer	3	JB: PhD Candidate<br>VK: Research Intern<br>EMB: Associate Professor
Gender	4	JB, VK, EMB, EKM: Cisgendered women
Experience and training	5	JB received training in qualitative interviewing at Radboud University Nijmegen. EMB received post-academic training in qualitative research methods at Erasmus University and Radboud University Nijmegen. EMB had extensive prior experience with interview and focus group studies.
Relationship with participants		
Relationship established	6	The interviewers had no relationship to participants prior to study commencement. Participants were contacted by JB via email.
Participant knowledge of the interviewer	7	Participants were informed about the interviewers' occupations as medical ethics researchers, at which institutions they were employed, and that they were interested in patient perspectives in order to guide ethical implementation of perfusion techniques. Participants knew the project was funded by a research consortium which investigates techniques to increase the quality and quantity of organs for transplantation. Personal opinions of interviewers were not shared.
Interviewer characteristics	8	Interviewers were aware of points of ethical controversy about perfusion techniques. Interviewers remained neutral during focus groups and attempted to present information in a balanced and objective way, and attempted to probe for participant views on these issues without steering their responses.
**Domain 2: Study Design**		
Theoretical framework		
Methodological orientation and Theory	9	Qualitative content analysis
Participant selection		

Sampling	10	Purposive recruitment was utilized to recruit transplant candidates and recipients from three patient groups (kidney, liver, heart). We attempted to recruit a diverse sample of participants considering age, gender, cultural group, education level, and clinical characteristics such as transplant status. For example, we ensured communications remained at a secondary school level. In selecting participants, maximum variation sampling was utilized within patient groups based on demographic and clinical characteristics.
Method of approach	11	Interested participants were emailed.
Sample size	12	32
Non-participation	13	Of the invitees, 7 dropped out: 3 were ill/unfit, 2 had scheduling difficulties, and 2 did not show up (no reason).
Setting		
Setting of data collection	14	Interviews were conducted online via a secure platform (Microsoft Teams).
Presence of non-participants	15	One family member of a participant joined a focus group to support the participant.
Description of sample	16	Between 04/2025 and 05/2025, five focus groups (1-2 per organ type) were conducted with 3-9 participants each. 32 participants were included: 16 liver patients, 13 kidney patients, and 3 heart patients. Demographics are presented in a table.
Data collection		
Interview guide	17	A semi structured interview guide and informative presentation about the techniques were utilized during focus groups. These materials were developed by the broader research team, with expertise spanning medical ethics, transplant medicine, healthcare psychology, and health technology assessment. 3 pilot focus groups were conducted with a total of 14 researchers unfamiliar with the study, and the materials were adapted after the pilot rounds. With each subsequent focus group, minor adjustments to materials were made to improve clarity. The information in the presentation and interview guide was presented as neutral, balanced, and objectively as possible.
Repeat interviews	18	No repeat focus groups were conducted.
Audio/visual recording	19	All focus groups were audio recorded, 3 were video recorded to ensure transcriptions reflected which participant was speaking. The recordings were auto-transcribed verbatim by Microsoft Teams, then manually corrected by 2 researchers each (JB and VK, MG, or JZ).
Field notes	20	Notes were taken during and after focus groups.
Duration	21	Focus groups lasted 100-120 min.

Data saturation	22	Data saturation was discussed, and was approached after 4 focus groups, after which 1 more focus group was carried out to confirm saturation across patient groups.
Transcripts returned	23	Transcripts were not returned to participants. Participants did receive an email to thank them for their participation, and were compensated 20 euros.
**Domain 3: analysis and findings**		
Data Analysis		
Number of data coders	24	The first transcript was open-coded by 5 researchers (JB, EMB, EKM, MS, JS) with backgrounds in medical ethics, healthcare psychology, and transplant medicine. The researchers met to discuss their interpretation/coding, and an initial codebook was developed. Three more transcripts were independently analyzed by two researchers (JB and EKM or VK) using open and closed coding. After another consensus discussion to finalize the codebook, one researcher (JB) coded the rest of the transcripts, discussing uncertainties in the interpretation with senior researchers (EKM and EMB). JB grouped and split up codes in an iterative process to develop themes and sub-themes, in consultation with senior researchers EKM and EMB.
Description of the coding tree	25	No description of the coding tree was provided. Codes were categorized into themes. Themes are presented and discussed in the Results section.
Derivation of themes	26	Themes were inductively derived from the data, but topics of discussion followed the interview guide, reflecting a partially directed approach.
Software	27	Atlas.ti version 24 was used for coding and analysis. Microsoft Excel was used to collate themes.
Participant checking	28	Participants did not provide feedback on the transcripts or findings.
Reporting		
Quotations presented	29	Quotations are presented in tables to illustrate the themes, and are identified by participant number.
Data and findings consistent	30	There is consistency between the data presented and the findings.
Clarity of major themes	31	Major themes are presented in separate sections of the results section.
Clarity of minor themes	32	Minor themes and diverse cases are presented within the sections on major themes, and in tables.

Developed from: Tong A, Sainsbury P, Craig J. Consolidated criteria for reporting qualitative research (COREQ): a 32-item checklist for interviews and focus groups. International Journal for Quality in Health Care. 2007. Volume 19, Number 6: pp. 349 – 357
