# Supplementary material for: Patient perspectives on the ethics and acceptability of perfusion techniques for organ transplantation: a qualitative study
Source: Transpl Int. 2026 Jul 8;39:16459. doi: 10.3389/ti.2026.16459 (PMC13388232; doi:10.3389/ti.2026.16459)
Supplement: Supplementary file 2 [file Supplementaryfile2.pdf]

Appendix 2. Focus Group Interview Guide

Perspectives of patients on organ perfusion

Date updated: 29 April 2025
Date translated from Dutch to English: 22 December 2025

Contents

Introduction (7 min) 2
- Agenda: Slides 2

Background (2 min) 3
- Background: Slides (2 min) 3

Machine Perfusion (MP) (15 min) 4
- MP: Slides (3 min) 4
- MP: General Knowledge and Attitudes 5
- MP: Current Applications – Perspectives and Acceptability 5

Normothermic Regional Perfusion (NRP) (25 min) 6
- NRP: Slides (5 min) 6
- NRP: General Knowledge and Attitudes 7
- NRP: Perspectives and Acceptability 7

Break (5–10 min) 8

Communication and Informed Consent (15 min) 8
- Communication Scenarios: Slides (2 min) 8
- Informed Consent: Preferences and Needs 8

Future (15 min) 9

Artificial Organs: Perspectives and Acceptability 9
- Artificial Organs: Slides (1 min) 9

Repair and Regeneration: Perspectives and Acceptability 10
- Repair and Regeneration: Slides (2 min) 10

Closing (5 min) 10

Introduction (7 min)

Welcome

Good morning/afternoon/evening and welcome everyone!
It's great that you have taken the time to participate in this focus group discussion, thank you very much for that. My name is [____], my colleague is [____], and we work as researchers for the [____].
We want to carry out an open, informal discussion, which is why I am addressing you informally.

Who are we and what are we going to do?

My colleague and I are researchers in the field of organ transplantation, but we are not doctors or medical researchers. We are particularly interested in the societal aspects. Therefore, we would like to hear your ideas about organ perfusion technology. This is a technology in transplantation to better preserve organs, which we will explain more about shortly. This focus group is one of [___]; we will combine the results of all the focus groups and use them for a scientific article.

You are invited because you are waiting for an organ transplant, or because you have been transplanted in the past. This means you have valuable experiences and ideas about what is important for patients.

Therefore, your input is valuable.

Introduction round

Let's first do a quick round of introductions. Can you all please share…

1) your name and where you come from?
2) how you came to join this focus group?
3) if you have experience participating in scientific research like this?
4) briefly tell us if you have been transplanted or are on the waiting list?

Agenda: Slides

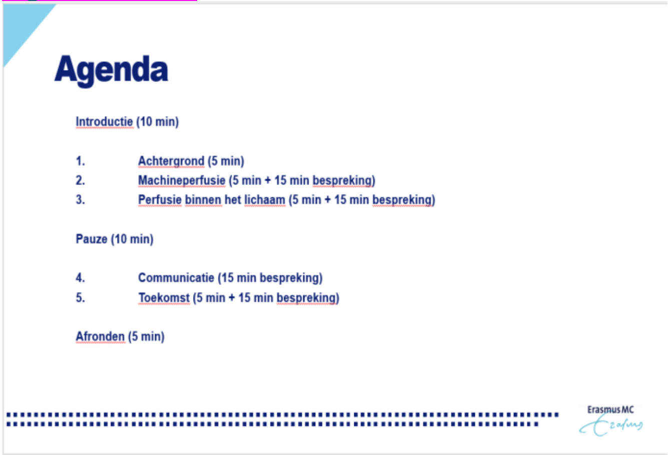

Here is our agenda for today. The meeting will last a maximum of 2 hours. We will take a 10-minute break after 1 hour.

We will discuss a number of different techniques. I will explain all of the techniques. We will do this one by one: I will speak for a few minutes, then we will have a discussion of about 15 minutes, and then we will move onto the next technique.

Discussion
In this discussion there are no right or wrong answers. Feel free to share your opinion, even if it differs from those of others. Please feel free to respond to one another — I will stay as much in the background as possible, because you are the experts. My colleagues and I are here to ask questions, listen, and guide the conversation where necessary.

Recording
As stated in the information letter that you received, we will audio-record and transcribe this discussion. Only my team and I will listen back to the recording. We will remove names and other identifiable information from the transcript.

If there are no objections, I will now start the recording.

[START RECORDER]

Background (2 min)

Background: Slides (2 min)

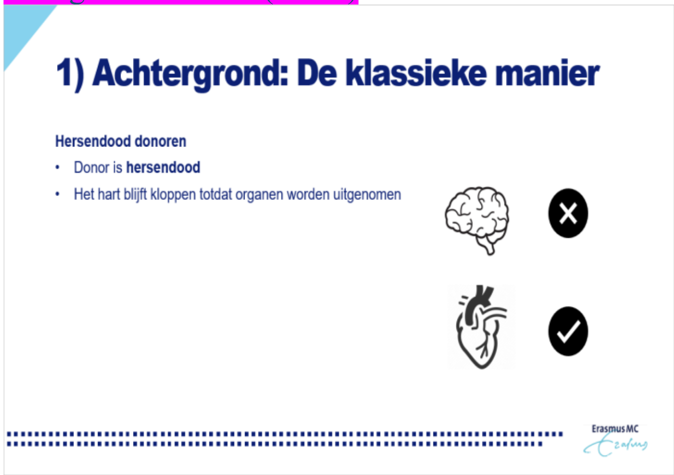

Every day, lives are saved through organ transplantation because donors are willing to donate their organs to patients who need them. Often these organs come from deceased donors.
In the past, only brain-dead donors were used. Brain-dead donors have lost all of their brain function and are therefore dead, but their heart continues to beat. This is ideal for organ donation because the organs continue to receive blood circulation.

[Next image] Unfortunately, there are not enough brain-dead donors. There is a shortage of organs, which means there are not enough transplantable organs available for all patients who need them.

[Next image] One solution is to allow donors who have died from cardiac death to donate their organs. A cardiac-death donor is a donor who has died because their heart has stopped beating. This means the organs no longer receive circulation, which causes damage to the organs. As a

result, new techniques are needed for cardiac-death donors to keep organs in good condition for transplantation.

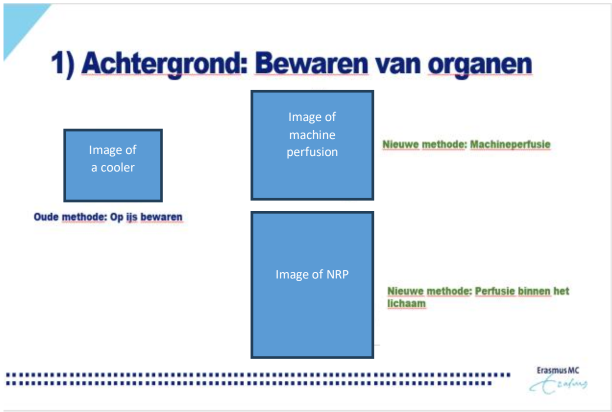

[Next slide] Typically, organs are stored in a cooler, also called "on ice." In recent years, new techniques have been developed to better preserve and transport organs: perfusion in a machine and perfusion inside the donor's body. This group discussion is about these new technologies.

Machine Perfusion (MP) (15 min)

MP: Slides (3 min)

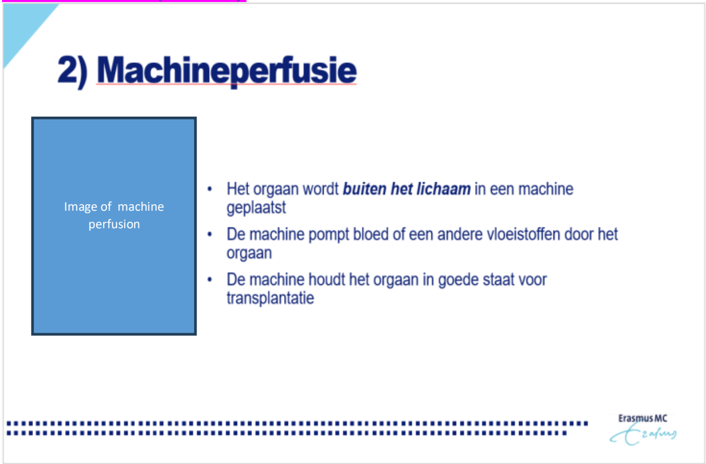

[Next slide] First, we will discuss machine perfusion. This is when an organ is placed in a machine outside the donor's body. There are machines for different organs: hearts, lungs, livers, and kidneys. The machine pumps blood or other fluids through the organ to keep it in good condition for transplantation. Organs are damaged when they do not receive blood circulation, and the machine helps to preserve them.

Machine perfusion can even be used during transport. We also call machine perfusion "on the pump."

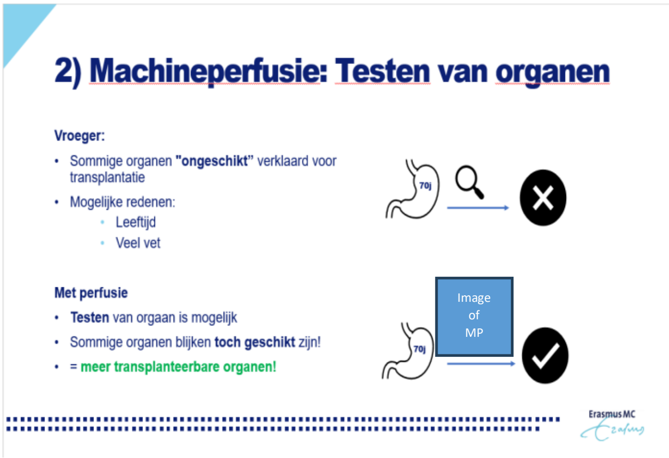

[Next slide] Perfusion also makes it possible to test the quality of organs.
In the past, organs were assessed with the naked eye by doctors to determine whether they were suitable for transplantation. Some organs that appeared to be of insufficient quality — for example due to age, excess fat, or damage — were therefore rejected for transplantation.

[Next image] Now, with perfusion, we can test these organs to see whether they still function well. This shows us that many organs that were previously considered unsuitable for transplantation are actually suitable for transplantation. This increases the number of available organs for transplantation.
Because machine perfusion is a new technique, the long-term outcomes are still unknown. However, we do know that short- and medium-term outcomes for these organs are good.

MP: General Knowledge and Attitudes

1) Have you heard of machine perfusion before?

2) What is your first impression of machine perfusion?

MP: Current Applications – Perspectives and Acceptability

3) What do you think about machine perfusion?
    - Cue: What do you see as the advantages?
    - Cue: What do you see as the disadvantages?
    - Cue: What do you think about organs that would previously have been rejected but are now used for transplantation through the use of perfusion?

4) If you had the choice, would you accept an organ that has been "on the pump"?
    - Cue: Under what circumstances would you be more willing to accept an organ that has been "on the pump"?
        - Cue: For example, if it meant you could receive an organ more quickly, would you accept it?
    - Cue: Under what circumstances would you be less willing to accept an organ that has been "on the pump"?

5) If you were to donate your organs after death, how would you feel about machine perfusion being used on your organs?

Normothermic Regional Perfusion (NRP) (25 min)

NRP: Slides (5 min)

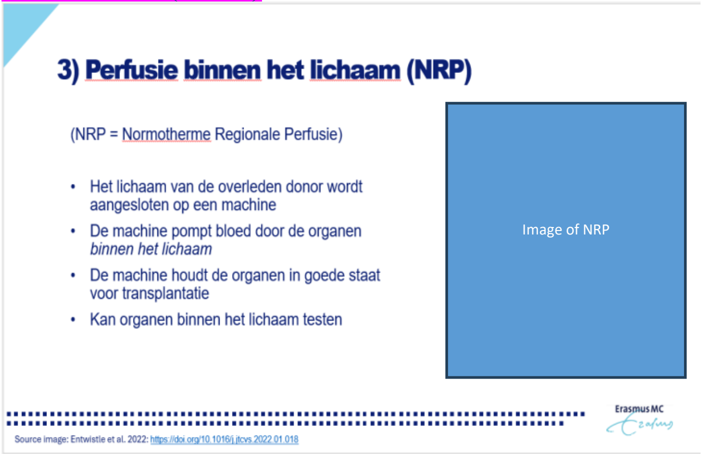

Now we will discuss the next technique: perfusion inside the body. This technique is similar to machine perfusion, except that it occurs within the donor's body. This is called NRP, which stands for normothermic regional perfusion, but we will simply call it "perfusion inside the body."

With this technique, after the heart has stopped beating and the donor has been declared dead, the body is connected to a machine. The machine pumps blood through the organs to keep them in good condition for transplantation.

[Next image] With perfusion inside the body, organs can also be tested to see whether they function properly, just like with machine perfusion.

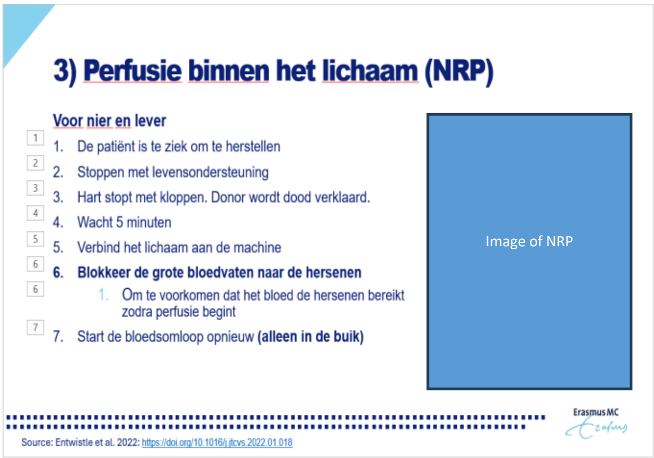

[Next slide] This technique is usually used for kidneys and the liver. I will walk you through the steps to make it clearer.

We begin with a patient who is unfortunately too ill to recover — for example, in a coma due to a drug overdose or heart attack. This patient has severe brain damage and therefore depends on life-support machines and cannot wake up again.

The doctor then has a conversation with the family. Together, a decision is made to withdraw life support.
Then, the heart stops beating, and the donor is declared dead.
Then, the doctors wait 5 minutes, in order to ensure that the heart does not start beating on its own.
Then the doctors connect the body to a machine.
Then the doctors block the large blood vessels to the brain. This is to prevent that the blood reaches the brain when perfusion begins.
Then the machine is turned on, and circulation is restarted. The circulation only reaches the belly, not the chest, brain, or any other part of the body.

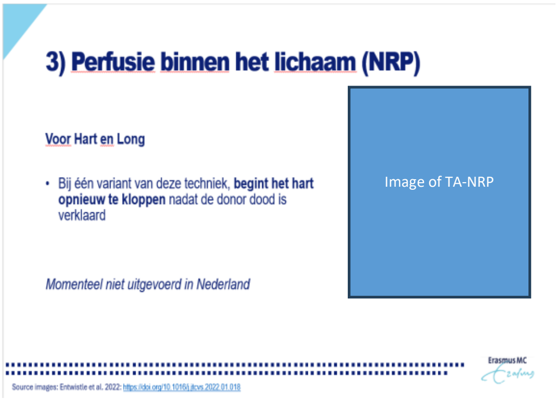

[Next slide] This technique can also be applied to the heart and lungs. In that case, blood circulation is restored to the chest as well as the belly. The heart then starts beating again after the donor has been declared dead. This variant of the technique is currently not performed in the Netherlands.

NRP: General Knowledge and Attitudes

6) What is your first impression of this technique?

NRP: Perspectives and Acceptability

7) What do you see as the advantages of this technique?
    - Cue: What do you think about the version of the technique where the heart is restarted?
    - Cue: What do you think about the step where the doctor blocks the blood vessels to the brain?

8) What do you see as the disadvantages of this technique?

9) If you had the choice, would you accept an organ that has undergone this technique?
    - Cue: Under what circumstances would you be more willing to accept an organ that has undergone this technique?

    - For example, if it meant you could receive an organ more quickly, would you accept it?
- Cue: Under what circumstances would you be less willing to accept an organ that has undergone this technique?

10) If you were to donate your organs after death, how would you feel about this technique being used on your organs?

Break (5–10 min)

We will now take a break and after the break we will discuss communication and informed consent. See you in 5-10 minutes.

[STOP VOICE RECORDER]

[START VOICE RECORDER]

Communication and Informed Consent (15 min)

Welcome back everyone. We are now going to discuss communication and informed consent. Now I will present three hypothetical procedures that could be used to provide information about machine perfusion and perfusion inside the body.

Communication Scenarios: Slides (2 min)

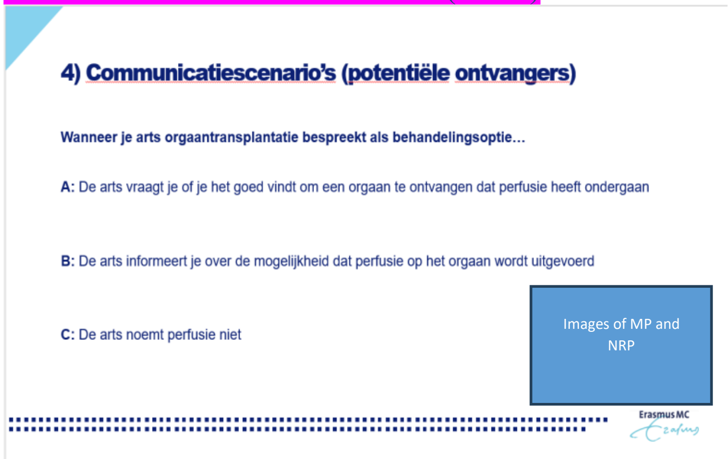

Imagine your doctor is discussing organ transplantation with you as a treatment option. There are three options for how you could receive information....

A) The doctor asks you if you are okay with receiving an organ that has undergone perfusion.
B) The doctor informs you about the possibility that perfusion has been performed on the organ.
C) The doctor does not mention perfusion

Informed Consent: Preferences and Needs

11) Which of these procedures (A, B, C) would you prefer and why?

12) Is your answer the same or different for perfusion inside the body versus perfusion outside the body in a machine?

If you chose the option where you want to receive information and/or want to give consent to perfusion:

13) What information do you need to make a good decision?

14) How would you like to receive this information?

[Next slide] Now imagine you were in the situation where you were informed by a doctor that the organs of your deceased relative were going to be donated. There are three options for how you could receive information about perfusion...

A) Information is given about perfusion, and you can decide if you are also open for perfusion alongside the normal donation procedure
B) Perfusion is described as part of the normal donation procedure
C) Perfusion is not mentioned

15) Which of these procedures (A, B, C) would you prefer and why?

Future (15 min)

Now we will talk about future possibilities.

Artificial Organs: Perspectives and Acceptability

Artificial Organs: Slides (1 min)

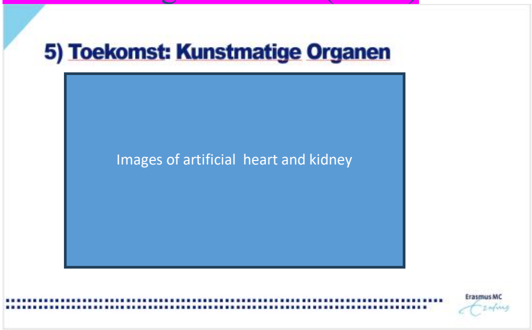

Alongside organ perfusion, other techniques are being developed, such as fully artificial organs.

16) Would you accept an artificial organ?

17) How do you compare artificial organs with perfused organs?
   - Cue: Which organ would you prefer, an artificial organ or a human organ from the perfusion machine?

Repair and Regeneration: Perspectives and Acceptability

Repair and Regeneration: Slides (2 min)

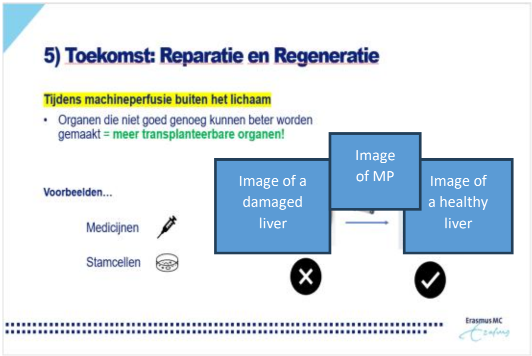

[Next slide] In the future, it may also be possible to repair and regenerate organs during machine perfusion outside the body. Organs that are still not good enough for transplantation, due to illness or damage, could be placed on the machine. Medicines or stem cells could then be added to make the organs as good as — or better than — standard organs. This would further increase the number of organs available for transplantation.

18) How do you compare "repaired" organs with artificial organs or standard organs?

19) Do these future procedures make a difference for you, in terms of how you feel about perfusion techniques, beyond what has already been discussed about organ perfusion?

20) Would you accept a repaired or regenerated organ?
    - Cue: Which organ would you prefer? A repaired organ, a standard organ from the perfusion machine, or an artificial organ?
    - Cue: Under what circumstances would you be more willing to accept a repaired organ?
    - Cue: Under what circumstances would you be less willing to accept a repaired organ?
    - Cue: Does it matter to you where the stem cells come from — animal, human, fetus, or yourself (the recipient)?
    - Cue: How do you feel about genetic modification of (animal) organs?

21) If you would receive information about any of the techniques we discussed today, is there anything specific in your values, religion, or culture that you think should be taken into account?

Closing (5 min)

We are now approaching the end of this group discussion.

22) Are there any topics we have not discussed that you consider important?

We hope that the results of this research will enable us to take an important first step in ethically developing and handling these new techniques in a way that aligns with the needs of people and

patients. Once the report with the results of this research is finished, in 6 to 12 months, I will send it to you.

I would like to sincerely thank you all for your time and contributions. As compensation, I will send each of you a €20 gift voucher by email. Thank you very much for your time. Have a nice rest of your day.

[STOP VOICE RECORDER]
